# Pyridoxine 5′-phosphate oxidase is correlated with human breast invasive ductal carcinoma development

**DOI:** 10.18632/aging.101908

**Published:** 2019-04-14

**Authors:** Weimin Ren, Wencai Guan, Jinguo Zhang, Fanchen Wang, Guoxiong Xu

**Affiliations:** 1 Research Center for Clinical Medicine, Jinshan Hospital, Fudan University, Shanghai 201508, China; 2 Department of Oncology, Shanghai Medical College, Fudan University, Shanghai 200032, China

**Keywords:** breast cancer, MALAT1, miR-216b, PNPO, vitamin B6

## Abstract

Pyridoxine 5′-phosphate oxidase (PNPO) is a converting enzyme for an active form of vitamin B6. This study aims to evaluate the biological function and the regulatory mechanism of PNPO in human breast invasive ductal carcinoma (IDC). We unveiled for the first time that PNPO was upregulated in patients with IDC and was correlated with the overall survival of patients with metastasis at the later stages. Suppression of PNPO inhibited breast cancer cell proliferation, migration, invasion and colony formation, arrested cell cycle at the G2/M phase and induced cell apoptosis. PNPO was positively correlated with lncRNA MALAT1 which was negatively correlated with miR-216b-5p. PNPO was down-regulated and up-regulated by miR-216b-5p mimics and inhibitors, respectively, in breast cancer cells. A microRNA response element was found in both PNPO and MALAT1 transcripts for miR-216b-5p and the dual-luciferase reporter assay confirmed the binding of these transcripts. Knockdown of MALAT1 resulted in an increase of miR-216b-5p and a decrease of PNPO mRNA, indicating a regulatory mechanism of competing endogenous RNAs. Taken together, these results reveal the biological function and a regulatory mechanism of PNPO, in which the MALAT1/miR-216b-5p/PNPO axis may be important in IDC development. Targeting this axis may have therapeutic potential for breast cancer.

## INTRODUCTION

Breast cancer is the most common malignant tumor and the second leading cause of cancer death in women worldwide [1]. The incidence rate of breast cancer keeps increasing in the USA and China [2]. Among all breast cancer cases, invasive ductal carcinoma (IDC) is the most common type of breast cancer that accounts for more than 70% of total diagnosed cases [3]. However, the mechanism of how IDC develops is unclear. Our previous study has shown that pyridoxine 5′-phosphate oxidase (PNP oxidase, PNPO) is overexpressed in human ovarian cancer and is a potential tumor progression marker [4]. It has been shown that PNPO is a converting enzyme for pyridoxal 5′-phosphate (PLP), an active form of vitamin B6, which serves as a co-factor for more than 140 enzymes that participate in many metabolic reactions such as amino acid, lipid, gluconeogenesis, and one-carbon, contributing to carcinogenesis-related events [5, 6]. PLP level was significantly associated with cancer risk [7]. PNPO activity affects PLP product and the biological behavior of ovarian cancer cells [4] and has an influence on colorectal cancer [8]. However, the biological function of PNPO in human breast IDC has not been studied yet. Furthermore, the correlation of PNPO expression with the breast cancer hormone therapy-related markers such as estrogen receptor (ER), progesterone receptor (PR) and human epidermal growth factor receptor-2 (HER2) is not clear. Also, a regulatory mechanism of PNPO is not fully understood.

Long non-coding RNAs (lncRNAs) are a category of non-coding RNAs, which have longer than 200 nucleotides in length with various regulatory or unknown functions. Metastasis-associated lung adenocarcinoma transcript 1 (MALAT1) is a well-studied lncRNA that plays an important role in tumorigenesis [9, 10]. Dysregulated expression of MALAT1 has been found in breast cancer [11] and is involved in a variety of signaling pathways, which makes it a star molecule with multiple functions [10, 12]. LncRNA-miRNA-mRNA interaction has been shown in cancers [13], including breast cancer [14]. Through the bioinformatics analyses, a microRNA response element (MRE) for miR-216b-5p is found in the 3′-untranslated region (3′-UTR) of PNPO and MALAT1 transcript. MiR-216b-5p plays a role in breast cancer served as a cancer suppressor [15] and participates in the most powerful interactions of metabolic pathways [16]. MALAT1/miR-216b-5p/PNPO may act as competing endogenous RNAs (ceRNAs) that affect the biological behavior of breast cancer cells and cancer progression.

In this study, the expression and function of PNPO were examined in patients with IDC and breast cancer cells. Particularly, a regulatory mechanism of the MALAT1/miR-216b-5p/PNPO axis was investigated. Whether PNPO expression is associated with clinicopathological features of IDC was also explored.

## RESULTS

### PNPO is overexpressed in IDC and correlated with clinicopathological features

**Figure 1:**
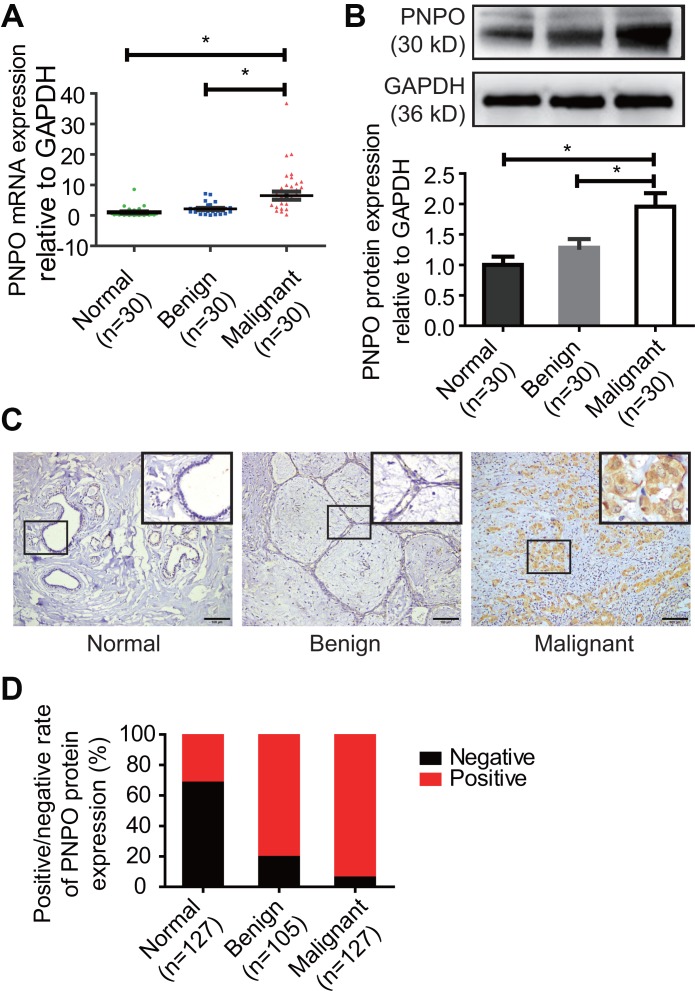
**PNPO expression in human breast tissues.** (**A**) PNPO mRNA expression was detected in breast tissues by qRT-PCR. (**B**) PNPO protein expression was detected in breast tissues by Western blot. Representative images and semi-quantitative analysis of the relative PNPO protein expression are shown in the upper and lower panel, respectively. (**C**) PNPO protein expression was detected in paraffin-embedded breast tissues by IHC. A picture in a frame is amplified. Brown color in a cell is considered positive staining. Original magnification, x200. Scale bar, 100 μm. (**D**) Quantified data of IHC. Normal, adjacent normal breast tissue; Benign, breast benign tumor (fibroadenomas); Malignant, breast malignant tumor (invasive ductal carcinoma); Negative, negative staining; positive, positive staining; n, number of cases. * *P* < 0.05.

The expression of PNPO mRNA and protein was higher in breast IDC (malignant tumor) tissues than adjacent normal breast tissues and fibroadenomas (benign tumor) tissues (Figure 1A and 1B). Immunohistochemistry (IHC) confirmed the overexpression of PNPO protein in patients with IDC (Figure 1C), which contained more positive cells (Figure 1D). To explore the clinical significance of PNPO expression in breast IDC, we divided the patients into two groups based on PNPO expression: low expression group (n = 56) and high expression group (n = 71). Analyses showed that the expression level of PNPO in IDC was not significantly correlated with clinicopathological features such as age, lymph node metastasis, tumor size, histological grade, and tumor stage (Supplementary Table 1). The expression of PNPO at the high and low levels was correlated with ER expression, but not PR, HER2, and a proliferation marker Ki-67, in breast cancer tissues (p = 0.04) (Supplementary Table 1).

### Serum PNPO is elevated in patients with IDC

**Figure 2:**
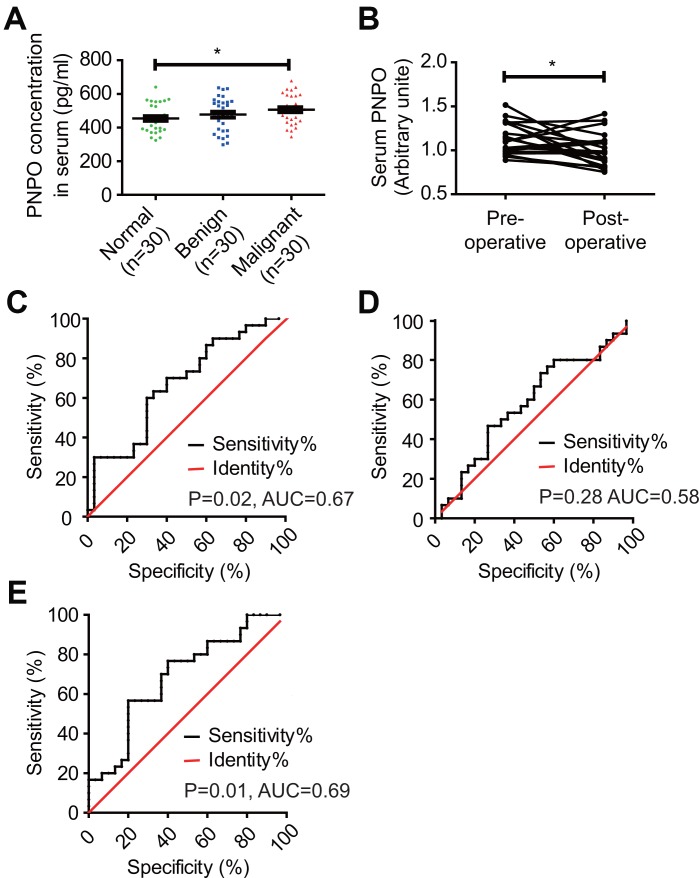
**Detection of serum PNPO concentration and calculation of receiver operating characteristic (ROC) curves.** (**A**) Measurement of PNPO concentration in the peripheral blood of women without tumor (Normal) and patients with fibroadenomas (Benign) or IDC (Malignant). * *P* < 0.05. (**B**) Relative PNPO levels in matched serum samples from IDC patients before (Pre-operative) and after (Post-operative) surgery. * *P* < 0.05. (**C**) ROC curves for the identification of breast IDC patients *vs.* non-cancer patients (n = 30, respectively) based on the concentration of PNPO in serum (*P* < 0.05). (**D**) ROC curves for the identification of breast IDC patients *vs.* non-cancer patients (n = 30, respectively) based on the concentration of COL5A1 in serum (*P* > 0.05). (**E**) ROC curves for the identification of breast IDC patients *vs.* non-cancer patients (n = 30, respectively) based on the concentration of PNPO and COL5A1 in serum (*P* < 0.05). AUC, the area under the curve; IDC, breast invasive ductal carcinoma; n, number of cases.

PNPO concentration was higher in patients with IDC than in women without tumor and patients with fibroadenoma (Figure 2A). The average concentrations of PNPO in the serum of IDC patients, the healthy controls, and patients with benign tumor were 506.37, 453.97, and 477.05 pg/ml, respectively. The relative levels of serum PNPO in matched samples were decreased after surgery in patients with IDC (P < 0.05) (Figure 2B), but no significant correlation was found between the level of serum PNPO and clinicopathological features (Supplementary Table 2). Next, the receiver operating characteristic (ROC) curves of serum PNPO was calculated. The ROC curve showed that an area under the curve (AUC) for PNPO is 0.67 with 95% confidence intervals (CIs) of 0.5330 to 0.8070 (P = 0.02) (Figure 2C). The concentration of serum COL5A1 in IDC patients, the benign tumor patients, and the healthy controls was 40.42, 43.08, and 42.24 μg/L, respectively. The AUC for COL5A1 was 0.58 with 95% CIs of 0.4346 to 0.7276 (P = 0.28) (Figure 2D). However, the AUC for PNPO combined with COL5A1 was 0.69 CIs of 0.553 to 0.8247 (P = 0.01) (Figure 2E).

### PNPO is correlated with the overall survival (OS) of IDC patients with metastasis at later stages

**Figure 3:**
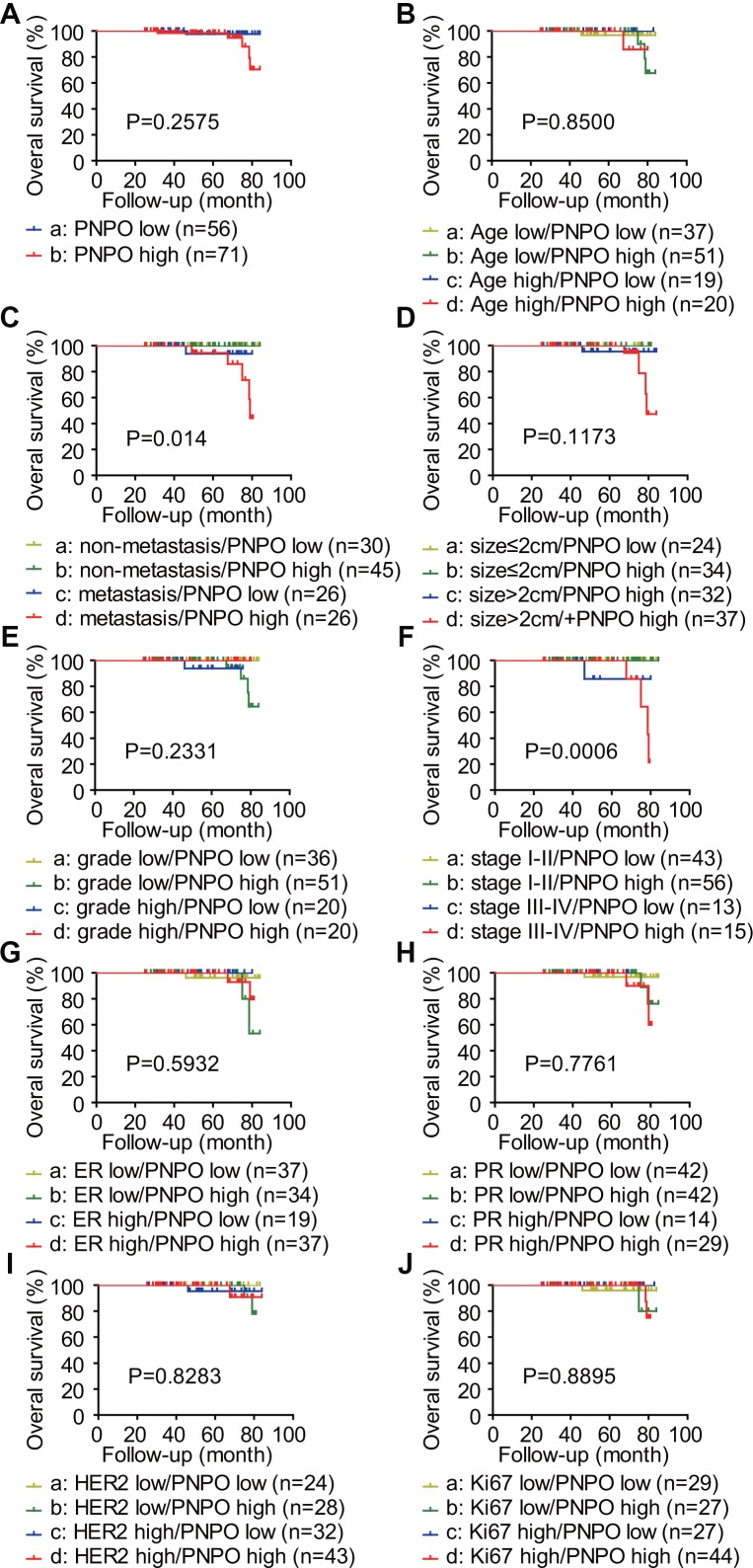
**Analysis of the correlation between the expression level of PNPO and the overall survival (OS) of patients with IDC.** PNPO expression was classified into two groups: PNPO low and PNPO high. The OS rate of 127 IDC patients was estimated by Kaplan-Meier analysis. The survival plots of IDC patients are shown. (**A**) Patients with low and high expression of PNPO. a *vs*. b: P=0.2575. (**B**) Patients with low and high expression of PNPO in age low (≤60 years) and high (>60 years) groups. a *vs*. b: P=0.6034; c *vs*. d: P=0.5127; a *vs*. c: P=0.5514; b *vs*. d: P=0.9694; a *vs*. d: P=0.5215; b *vs*. c: P=0.5442. (**C**) Patients with low and high expression of PNPO in non-metastasis and metastasis groups. a *vs* s. b: P=1.0000; c *vs*. d: P=0.3299; a *vs*. c: P=0.2024; b *vs*. d: P=0.0128; a *vs*. d: P=0.0385; b *vs*. c: P=0.1782. (**D**) Patients with low and high expression of PNPO in small (≤2 cm) and large (>2 cm) tumor groups. a *vs.* b: P=1.0000; *vs.* d: P=0.3339; a *vs.* c: P=0.3527; b *vs.* d: P=0.0459; a *vs.* d: P=0.1953; b *vs.* c: P=0.3173. (**E**) Patients with low and high expression of PNPO in low grade (1+2) and high grade (3) groups. a *vs.* b: P=0.1169; c *vs.* d: P=0.4070; a *vs.* c: P=0.2024; b *vs.* d: P=0.2542; a *vs.* d: P=1.0000; b *vs.* c: P=0.6708. (**F**) Patients with low and high expression of PNPO in early (I+II) and later (III+IV) stage groups. a *vs.* b: P=1.0000; c *vs.* d: P=0.5600; a *vs.* c: P=0.0253; b *vs.* d: P=0.0009; a *vs.* d: P=0.0112; b *vs.* c: P=0.0223. (**G**) Patients with low and high expression of PNPO in ER low and high expression groups. a *vs.* b: P=0.4315; c *vs.* d: P=0.4520; a *vs.* c: P=0.4328; b *vs.* d: P=0.4912; a *vs.* d: P=0.7461; b *vs.* c: P=0.3919. (**H**) Patients with low and high expression of PNPO in PR low and high expression groups. a *vs.* b: P=0.6074; c *vs.* d: P=0.6547; a *vs.* c: P=0.5514; b *vs.* d: P=0.4993; a *vs.* d: P=0.3594; b *vs.* c: P=1.0000. (**I**) Patients with low and high expression of PNPO in HER2 low and high expression groups. a *vs.* b: P=0.4549; c *vs.* d: P=0.9261; a *vs.* c: P=0.3404; b *vs.* d: P=0.8380; a *vs.* d: P=0.3404; b *vs.* c: P=0.9143. (**J**) Patients with low and high expression of PNPO in less (≤10) and more (>10) Ki-67 positive groups. a *vs.* b: P=0.9372; c *vs.* d: P=0.6048; a *vs.* c: P=0.4096; b *vs.* d: P=0.7686; a *vs.* d: P=0.9474; b *vs.* c: P=0.3711.

PNPO expression was classified into two groups: PNPO low and PNPO high, based on the staining index (SI) by IHC. The OS rate of 127 IDC patients was estimated by Kaplan-Meier analysis. Figure 3 showed the OS of patients with IDC between different groups: low expression of PNPO *vs*. high expression of PNPO, age low (≤60 years) *vs*. age high (>60 years), non-metastasis *vs*. metastasis, small tumor size (≤2 cm) *vs*. large tumor size (>2 cm), low grade (1+2) *vs*. high grade (3), early stage (I+II) *vs*. later stage (III+IV), low expression of ER *vs*. high expression of ER, low expression of PR *vs*. high expression of PR, low expression of HER2 *vs*. high expression of HER2, less Ki-67 positive (≤10) *vs*. more Ki-67 positive (>10). Kaplan-Meier analysis of the OS of IDC patients showed that patients with high PNPO expression were related to metastasis and clinical stages. Patients with high PNPO expression had higher mortality compared to those with low PNPO expression status (P < 0.05) (Figure 3C and 3F). Subgroup analyses for OS with tumor-related death (TD), hazard ratio (HR), and 95% confidence intervals (CIs) were conducted by the univariate analysis using the Cox proportional hazard regression model. No significant association of OS between PNPO expression and clinicopathological features was observed in patients with IDC (Supplementary Table 3). Furthermore, multivariate analyses with factors being significantly associated with the outcome in Western blot and Log-rank (Mantel-Cox) analyses were also applied. No significant association was observed either (Supplementary Table 4).

### PNPO expression is correlated with progesterone receptor expression

**Figure 4:**
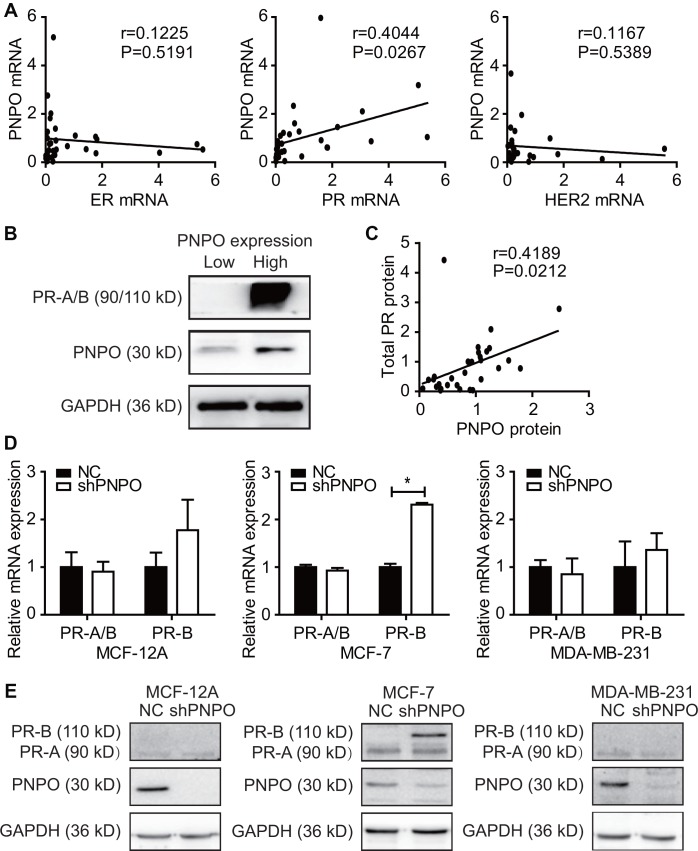
**Correlation of PNPO expression with progesterone receptor (PR) expression.** (**A**) Detection of the correlation of PNPO mRNA expression with the expression of ER, PR and HER2 mRNA in breast IDC tissues by qRT-PCR (n = 30 each). (**B**) Expression of PR and PNPO proteins in IDC tissues of two patients with a low level of PNPO (left sample) and the high level of PNPO (right sample) detected by Western blot. (**C**) Correlation of PNPO protein expression with PR protein expression in IDC tissues (n =30). (**D**) Effect of PNPO knockdown by shRNA on PR mRNA expression detected by qRT-PCR. Breast non-cancerous cells (MCF-12A) and cancerous cells (MCF-7 and MDA-MB-231) were treated with PNPO-shRNA (shPNPO) and its negative control (NC). A total PR (PR-A/B) and a B-type PR (PR-B) were detected by qRT-PCR (n = 3). * *P* < 0.05. (**E**) Effect of PNPO knockdown by shRNA on PR protein expression detected by Western blot in MCF-12A, MCF-7, and MDA-MB-231 cells.

In IDC tissues, a positive correlation was found between PNPO and PR (P = 0.0267), but not ER (P = 0.5191) or HER2 (P = 0.5389), at the mRNA level (Figure 4A). Two IDC samples obtained from two individual patients who had either low or high expression of PNPO mRNA were selected to confirm the correlation between PNPO and PR protein expression (Figure 4B). Indeed, a positive correlation between PNPO and PR was verified at the protein level (P = 0.212) (Figure 4C). Next, the expression of PNPO protein in five different breast cell lines including hormone-sensitive and -insensitive cells was examined by Western blot. All examined breast cells, including non-cancerous cells MCF-12A (ER-/PR-/HER2-) and cancerous cells MCF-7 (ER+/PR+/HER2-), MDA-MB-231 (ER-/PR-/HER2-), SK-BR-3 (ER-/PR-/HER2+), and BT-474 (ER+/PR+/HER2+). were PNPO-positive (Supplementary Figure 1). Knockdown of PNPO by shRNA increased the expression of a B-type of PR (PR-B isoform) mRNA (Figure 4D) and protein (Figure 4E) in hormone-sensitive MCF-7 cells. By contrast, there was an undetectable level of PR in hormone-insensitive cells MDA-MB-231 and MCF-12A.

### Knockdown of PNPO inhibits breast cancer cell proliferation, migration, invasion and colony formation

**Figure 5:**
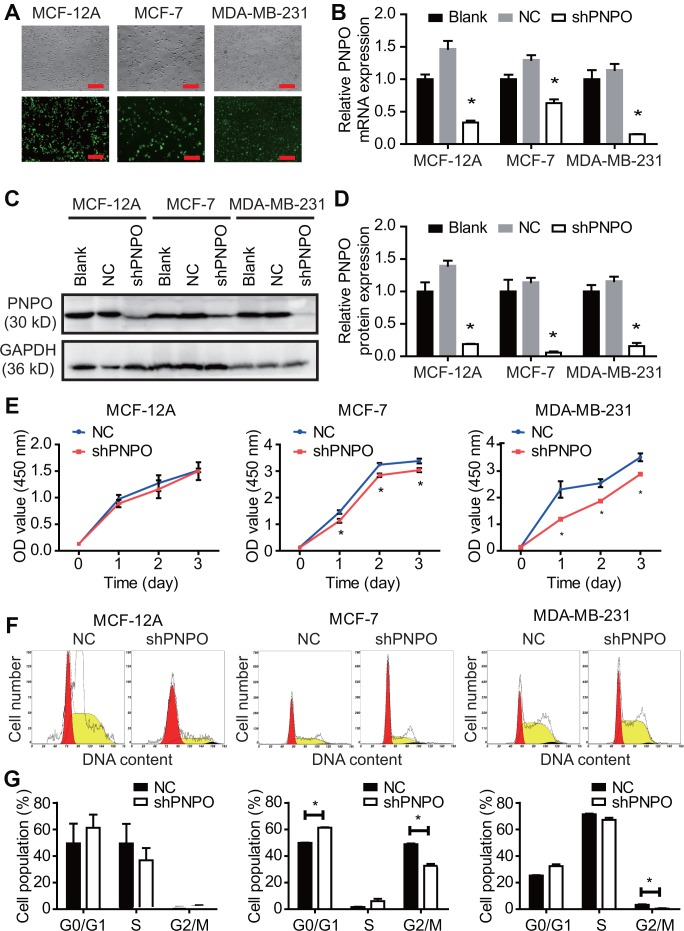
**The effect of PNPO on breast cell proliferation.** (**A**) The efficiency of lentiviruses infection with GFP and PNPO-shRNA in breast non-cancerous cells (MCF-12A) and cancerous cells (MCF-7 and MDA-MB-231) detected by immunofluorescence microscopy. Scale bar, 500 μm. (**B**) Effect of PNPO knockdown by PNPO-shRNA on PNPO mRNA expression in MCF-12A, MCF-7, and MDA-MB-231 cells detected by qRT-PCR. (**C**) Effect of PNPO knockdown by PNPO-shRNA on PNPO protein expression in MCF-12A, MCF-7, and MDA-MB-231 cells detected by Western blot. (**D**) Histograms show the semi-quantitative analyses of the gels from (**C**) after densitometry. (**E**) Effect of PNPO knockdown by PNPO-shRNA on cell viability in MCF-12A, MCF-7, and MDA-MB-231 cells detected by the CCK-8 assay. (**F**) Effect of PNPO knockdown by PNPO-shRNA on cell cycle in MCF-12A, MCF-7, and MDA-MB-231 cells detected by flow cytometry. (**G**) Histograms show the semi-quantitative analyses of the cell population in cell cycle phases. Blank, non-infected cells; NC, negative control of shRNA; shPNPO, PNPO-shRNA. n = 3; * *P* < 0.05.

The effect of PNPO on cellular behavior was examined by the loss-of-function approach. Immunofluorescence microscopy showed the efficiency of lentiviruses infection with GFP and PNPO-shRNA in MCF-12A, MCF-7, and MDA-MB-231 cells and more than 90% of cells were GFP-positive (Figure 5A). PNPO was significantly knocked down by PNPO-shRNA at the mRNA (Figure 5B) and protein levels (Figure 5C and 5D). Using a CCK8 assay, a decrease of cell viability was observed in breast cancer cells MCF-7 and MDA-MB-231 after PNPO-shRNA infection (Figure 5E). Flow cytometry showed that PNPO knockdown increased G0/G1 phase in MAF-7 cells and decreased G2/M phase in MCF-7 and MDA-MB-231 cells (Figure 5F and 5G).

**Figure 6:**
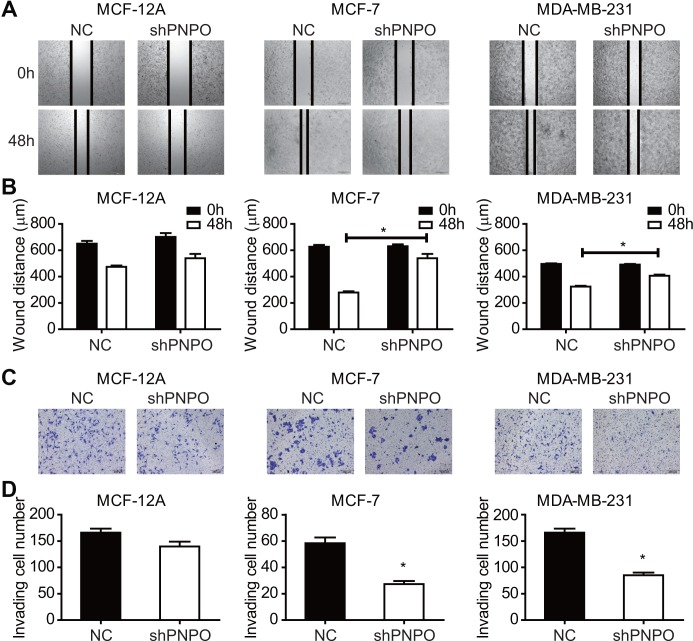
**The effect of PNPO on breast cell migration and invasion.** (**A**) Effect of PNPO knockdown by PNPO-shRNA on cell migration in MCF-12A, MCF-7, and MDA-MB-231 cells assessed by the wound-healing assay (Original magnification, x500). (**B**) Histograms show the quantitative analyses of wound distance at 0 and 48 h post-scratch (n = 3). (**C**) Effect of PNPO knockdown by PNPO-shRNA on cell invasion in MCF-12A, MCF-7, and MDA-MB-231 cells detected by the Transwell assay (Original magnification, x200). (**D**) Histograms show the number of invading cells (n = 3). NC, negative control of shRNA; shPNPO, PNPO-shRNA. * *P* < 0.05.

The effect of PNPO knockdown on cell migration and invasion was evaluated by the wound-healing and Transwell assays, respectively. The growth gap was assessed by measuring the wound distance (Figure 6A). The wound-healing was significantly slower in MCF-7 and MDA-MB-231 cells after PNPO knockdown (Figure 6B). In a Transwell assay, a reduced number of invading cells was observed in MCF-7 and MDA-MB-231 cells after PNPO knockdown for 48 h (Figure 6C and 6D).

**Figure 7:**
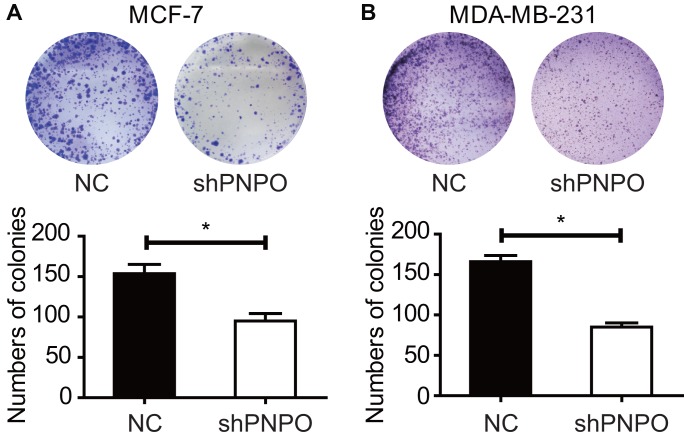
**The effect of PNPO on the colony formation in breast cancer cells.** (**A**) Detection of colonies in MCF-7 cells. (**B**) Detection of colonies in MDA-MB-231 cells. Upper panel, pictures of colony formation; lower panel, quantification of colony number. NC, negative control of shRNA; shPNPO, PNPO-shRNA. n = 3; * *P* < 0.05.

Knockdown of PNPO affected colony formation. The number of colonies was reduced after PNPO-shRNA infection in MCF-7 (Figure 7A) and MDA-MB-231 (Figure 7B) cells.

### Knockdown of PNPO promotes breast cancer cell apoptosis

**Figure 8:**
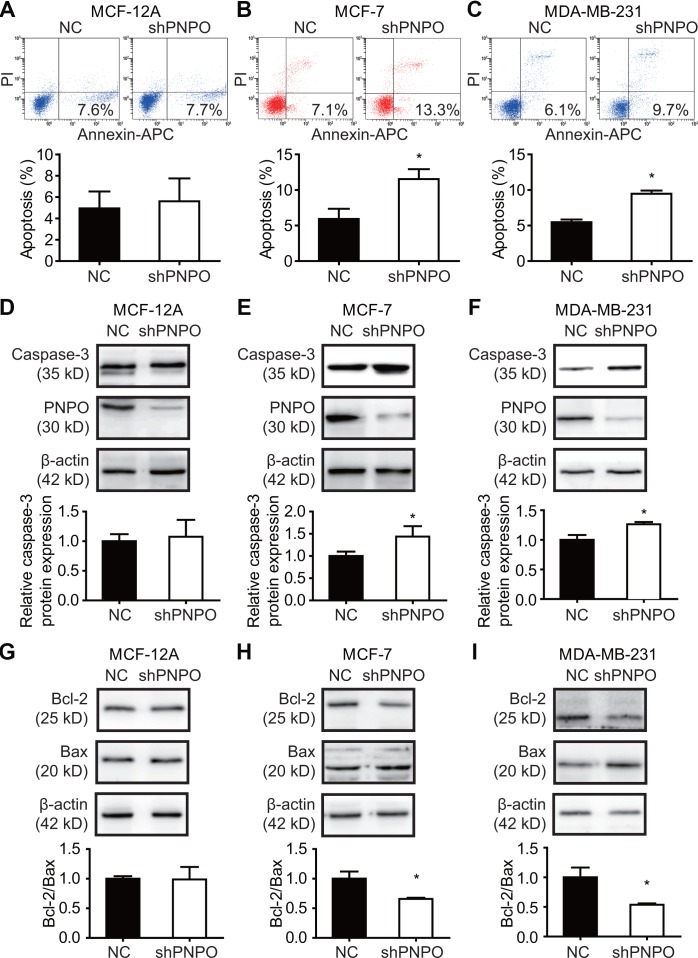
**The effect of PNPO on breast cell apoptosis.** (**A**–**C**) Apoptotic cells were detected by flow cytometry after PNPO knockdown in MCF-12A (**A**), MCF-7 (**B**), and MDA-MB-231 (**C**) cells. (**D**–**F**) Expression of caspase-3 and PNPO protein after PNPO knockdown in MCF-12A (**D**), MCF-7 (**E**), and MDA-MB-231 (**F**) cells detection by Western blot. (**G**–**I**) Expression of Bcl-2 and Bax protein after PNPO knockdown in MCF-12A (**G**), MCF-7 (**H**), and MDA-MB-231 (**I**) cells detection by Western blot. Histograms show the quantitative analyses. NC, negative control of shRNA; shPNPO, PNPO-shRNA. n =3; * *P* < 0.05.

Flow cytometry showed that the number of early apoptotic cells was increased in MCF-7 and MDA-MB-231 cells, not in MCF-12A cells, after PNPO knockdown compared with NC cells (Figure 8A–8C). After PNPO knockdown was confirmed, an increased caspase-3 was detected only in MCF-7 and MDA-MB-231 cells, not in MCF-12A cells (Figure 8D–8F). Further, a decreased ratio of anti-apoptotic factor Bcl-2 over pro-apoptotic factor Bax was found in MCF-7 and MDA-MB-231 cells, not in MCF-12A cells, after PNPO knockdown compared with NC cells (Figure 8G–8I).

### MALAT1 is correlated with PNPO and miR-216b-5p

**Figure 9:**
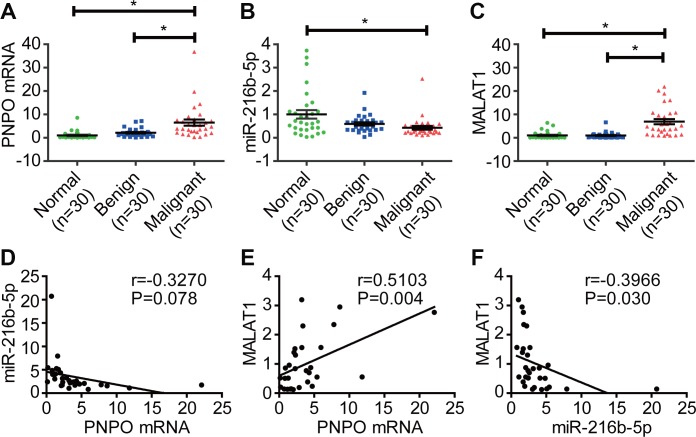
**Correlation of PNPO with miR-216b-5p and MALAT1 in breast tissues.** (**A**) Detection of PNPO mRNA in breast tissues by qRT-PCR. (**B**) Detection of miR-216b-5p expression in breast tissues by qRT-PCR. (**C**) Detection of MALAT1 expression in breast tissues by qRT-PCR. (**D**) Correlation of miR-216b-5p expression with PNPO expression in patients with IDC. (**E**) Correlation of MALAT1 expression with PNPO expression in patients with IDC. (**F**) Correlation of MALAT1 expression with miR-216b-5p expression in patients with IDC. IDC, invasive ductal carcinoma Normal, adjacent normal breast tissue; Benign, breast benign tumor (fibroadenomas); Malignant, breast malignant tumor (IDC); n = 30; * *P* < 0.05.

The expression of PNPO mRNA (Figure 9A), miR-216b-5p (Figure 9B), and MALAT1 (Figure 9C) in breast tissues was detected by qRT-PCR. An increase of PNPO mRNA and MALAT1, while a decrease of miR-216b-5p, was observed in IDC tissues. PNPO mRNA tended to be negatively correlated with miR-216b-5p expression (P = 0.078) (Figure 9D) but positively correlated with MALAT1 expression (P = 0.004) (Figure 9E) in patients with IDC. Further, MALAT1 expression was negatively correlated with miR-216b-5p expression in patients with IDC (P = 0.03) (Figure 9F).

### PNPO is directly regulated by miR-216b-5p in breast cancer cells

**Figure 10:**
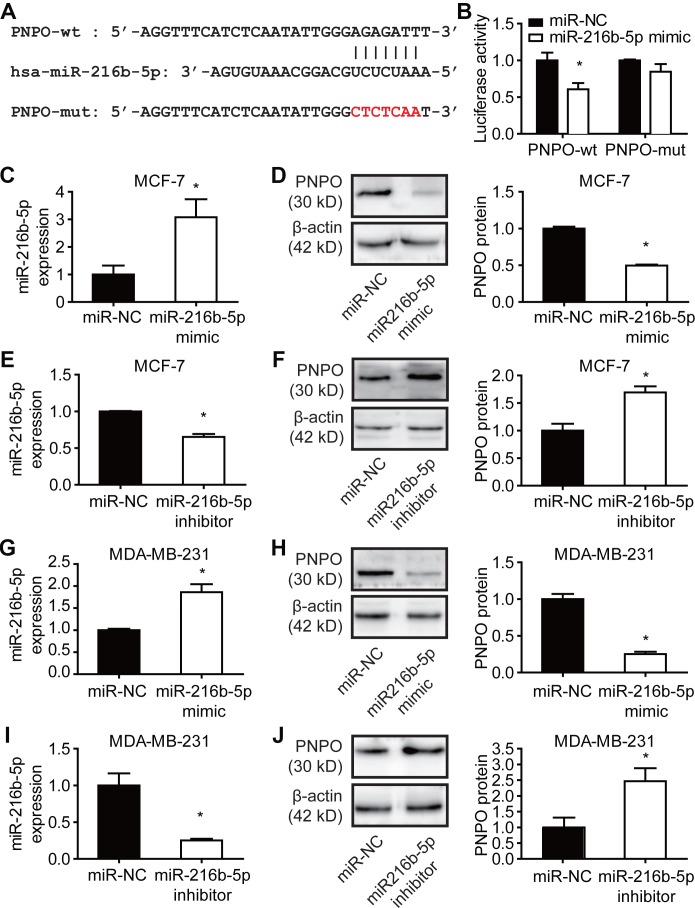
**Effect of miR-216b-5p on PNPO expression in breast cancer cells.** (**A**) Identification of the sequence of binding sites between PNPO mRNA 3’-UTR and miR-216b-5p. PNPO-wt, wild-type PNPO; PNPO-mut, mutated PNPO. (**B**) Detection of luciferase activity by the dual-luciferase reporter assay in HEK-293T cells. (**C**) Detection of miR-216b-5p by qRT-PCR in MCF-7 cells after miR-216b mimics treatment. (**D**) Effect of miR-216b mimics on PNPO expression detected by Western blot (left) and semi-quantification (right) in MCF-7 cells. (**E**) Detection of miR-216b-5p by qRT-PCR in MCF-7 cells after miR-216b inhibitors treatment. (**F**) Effect of miR-216b inhibitors on PNPO expression detected by Western blot (left) and semi-quantification (right) in MCF-7 cells. (**G**) Detection of miR-216b-5p by qRT-PCR in MDA-MB-231 cells after miR-216b mimics treatment. (**H**) Effect of miR-216b mimics on PNPO expression detected by Western blot (left) and semi-quantification (right) in MDA-MB-231 cells. (**I**) Detection of miR-216b-5p by qRT-PCR in MDA-MB-231 cells after miR-216b inhibitors treatment. (**J**) Effect of miR-216b inhibitors on PNPO expression detected by Western blot (left) and semi-quantification (right) in MDA-MB-231. miR-NC, negative control of miRNA. n = 3; * *P* < 0.05.

Using online databases of DIANA (http://carolina.imis.athena-innovation.gr/diana_tools/web/index.php?r=site%2Findex), miRanda (http://www.microrna.org/microrna/home.do), and Starbase (http://starbase.sysu.edu.cn) for targeting prediction, we found that both PNPO and MALAT1 contain a potential binding site for miR-216b-5p. An MRE sequence was identified in the PNPO mRNA 3′-UTR (PNPO-wt) which was mutated from AGAGATT to CTCTCAA (PNPO-mut) (Figure 10A). The dual-luciferase reporter assay showed that the luciferase activity in HEK-293T cells was reduced after 48 h post-transfection with PNPO-wt plasmid and miR-216b-5p mimics and the interaction between PNPO and miR-216b-5p was abolished in cells transfected with PNPO-mut plasmid (Figure 10B). An increase of miR-216b-5p was confirmed after treating MCF-7 cells with mimics (Figure 10C). Treatment of miR-216b mimics resulted in a decrease of PNPO protein in MCF-7 cells (Figure 10D). Treatment of miR-216b inhibitors resulted in a decrease of miR-216b-5p (Figure 10E) and an increase of PNPO protein (Figure 10F) in MCF-7 cells. The similar effect of mimics and inhibitors of miR-216b-5p was observed in MDA-MB-231 cells (Figure 10G–10J).

### PNPO is regulated by MALAT1 via competitive binding for miR-216b-5p in breast cancer cells

**Figure 11:**
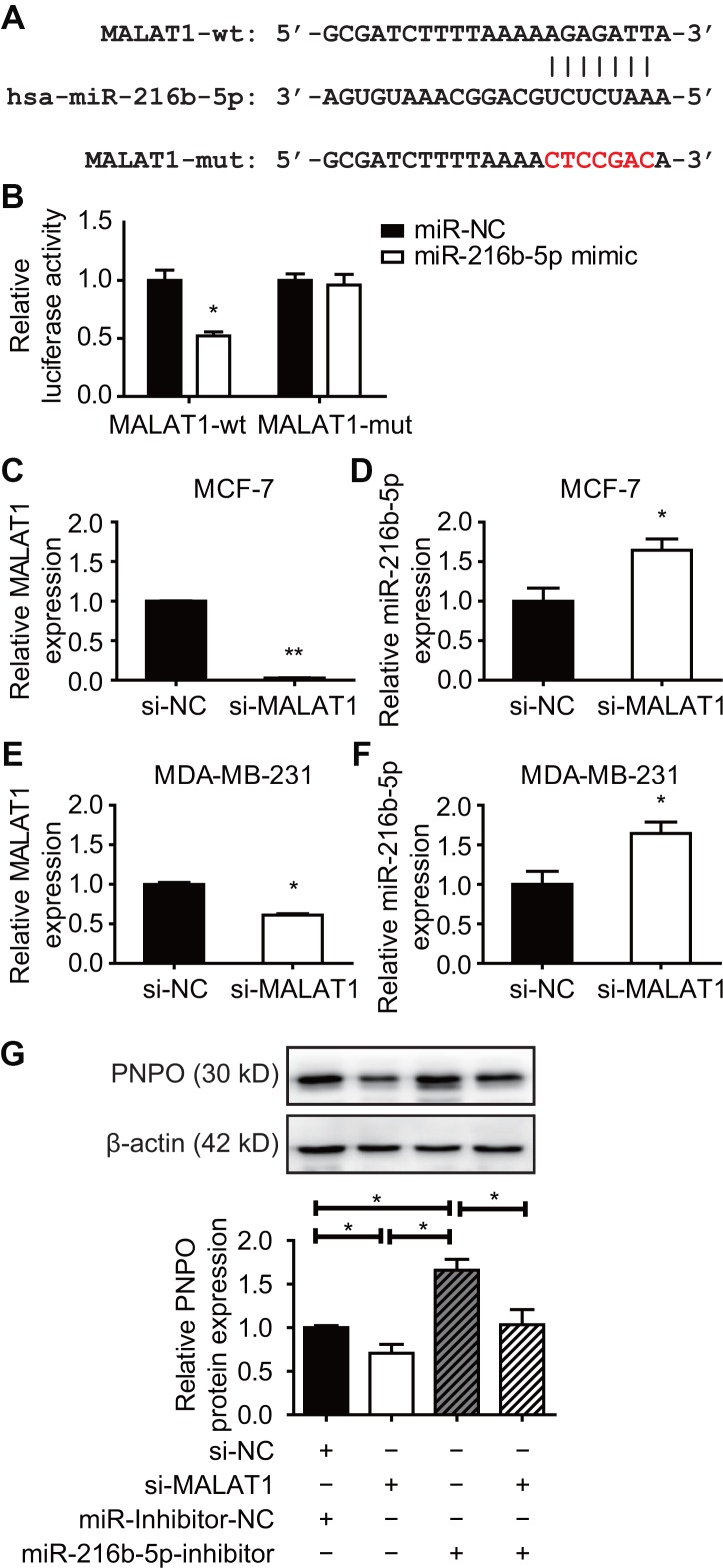
**Effect of MALAT1 on PNPO and miR-216b-5p expression in breast cancer cells**. (**A**) Identification of the sequence of binding sites between MALAT1 and miR-216b-5p. MALAT1-wt, wild-type MALAT1; MALAT1-mut, mutated MALAT1. (**B**) Detection of luciferase activity by the dual-luciferase reporter assay in HEK-293T cells. (**C**) Detection of MALAT1 expression by qRT-PCR in MCF-7 cells after si-MALAT1 treatment. (**D**) Detection of miR-216b-5p expression by qRT-PCR in MCF-7 cells after si-MALAT1 treatment. (**E**) Detection of MALAT1 expression by qRT-PCR in MDA-MB-231 cells after si-MALAT1 treatment. (**F**) Detection of miR-216b-5p expression by qRT-PCR in MDA-MB-231 cells after si-MALAT1 treatment. (**G**) Effect of miR-216b-5p on MALAT1-mediated PNPO expression. HEK-293T cells were treated with si-NC plus miR-inhibitor-NC, si-MALAT1 along, miR-216b-5p along, or si-MALAT1 plus miR-216b-5p for 48 h. The expression of PNPO protein was detected by Western blot (upper panel) and semi-quantification (lower panel). miR-NC, negative control of miRNA; miR-inhibitor-NC, negative control of miR-inhibitor; si-NC, negative control of siRNA; si-MALAT1, MALAT1-siRNA. n = 3; * *P* < 0.05.

An MRE sequence was identified in the MALAT1 transcript (MALAT1-wt) which was mutated from AGAGATT to CTCCGAC (MALAT1-mut) (Figure 11A). The dual-luciferase reporter assay showed a reduced luciferase activity in HEK-293T cells transfected with MALAT1-wt plasmid and miR-216b-5p mimics for 48 h and the interaction between MALAT1 and miR-216b-5p was abolished in cells transfected with MALAT1-mut plasmid (Figure 11B). The expression of MALAT1 was knocked down (Figure 11C), while miR-216n-5p was increased (Figure 11D), after MALAT1-siRNA transfection in MCF-7 cells. The same effect of MALAT1-siRNA was observed in MDA-MB-231 cells (Figure 11E and 11F). Next, a rescue experiment was applied. HEK-293T cells were treated with si-NC plus miR-inhibitor-NC, si-MALAT1 along, miR-216b-5p along, or si-MALAT1 plus miR-216b-5p for 48 h and PNPO protein was detected by Western blot. Knockdown of MALAT1 resulted in a decreased of PNPO expression, whereas the inhibition of miR-216b-5p resulted in an increase of PNPO expression (Figure 11G). The effect of MALAT1-siRNA on PNPO expression was slightly abolished in the presence of miR-216b-5p inhibitors. These data suggest that PNPO in breast cancer cells is regulated at least in part through the MALAT1/miR-216b-5p/PNPO axis.

## DISCUSSION

The present study demonstrated for the first time that PNPO was upregulated in IDC at mRNA and protein levels and correlated with the overall survival of patients with breast cancer. Knockdown of PNPO affected breast cancer cell behavior and was associated with PR expression. Furthermore, the ceRNA mechanism in the regulation of PNPO was found in breast cancer cells.

PNPO is a key enzyme oxidizing pyridoxine 5′-phosphate and pyridoxamine 5′-phosphate into the active form of vitamin B6 PLP [17]. Dysregulated PNPO has been reported previously. For instance, a gene array study reported that PNPO is upregulated in colorectal cancer and can predict overall survival [8] and is one of the important molecules involved in ovarian cancer development [4]. These data indicate that PNPO may play a role in tumorigenesis. The current study unveiled an overexpression of PNPO in patients with IDC at the mRNA and protein levels. Based on the primary tumor, lymph node, and metastasis (TNM) classification [18], the correlation of PNPO expression with clinical characteristics was analyzed. We found that the expression level of PNPO was correlated with IDC metastasis at the later stage. Furthermore, the concentration of serum PNPO was increased in breast cancer patients, suggesting that PNPO may serve as a serum marker for IDC detection.

The overexpression of PNPO was observed in patients with IDC. Although the log-rank test showed that OS with the factors of metastasis and clinical stage was influenced by PNPO expression level, the univariate and multivariate analyses for the outcome of OS showed less strength of the link between PNPO and clinical prognosis. These data suggest that PNPO may be a progression marker rather than an independent prognostic factor. Because the sample size used in the current study is relatively small and the time of observation for OS is short, these outcomes may not really reflect the clinical importance of PNPO. Accumulated data analyses will be needed in the future.

The current study using a loss-of-function approach examined the biological functions of PNPO. Decreasing PNPO may suppress tumor-initiating potential. We found that knockdown of PNPO resulted in a decrease of breast cancer cell proliferation, migration, invasion, and colony formation, arrested cell cycle at the G2/M phase, and induced cell apoptosis. These data may implicate in the application of small molecules targeting the PNPO.

Patients with ER-positive and PR-positive breast cancer are responsible for hormone-therapies [19]. However, there are limited treatment options for triple-negative breast cancer (ER-/PR-/HER2-) compared with triple-positive one [20]. In human IDC tissue study, we observed the positive correlation between PNPO and ER (P = 0.040), not PR (P = 0.061), at protein level (n=127) detected by IHC and the positive correlation between PNPO and PR at mRNA and protein levels (both n = 30; P < 0.05) detected by qRT-PCR and Western blot, respectively. The different results observed in this study were most likely due to the utilization of the different methods and small sample size, and therefore, an increase in the sample size may be needed in the subsequent study. The current study also examined the effect of PNPO on PR expression in hormone-sensitive and -insensitive breast cancer cell lines. It has been shown that MCF-12A and MDA-MB-231 are ER-/PR-/HER2- cell lines, whereas MCF-7 is ER+/PR+/HER2- cell line [21]. Most interestingly, we found that knockdown of PNPO increased the expression of PR-B isoform in MCF-7 cells, not in MDA-MB-231 cells, suggesting that PNPO may influence the hormone sensitivity of breast cancer cells. Because of the vast heterogeneity in breast cancer and because the regulation of PR due to PNPO knockdown in MCF-7 cells remains unknown, along with this line, further study may be needed.

The isoforms of PR-A and PR-B may have different physiological functions [22]. The expression ratio of PR-A/PR-B is frequently disrupted during breast cancer progression and imbalanced PR-A/PR-B expression in breast cancer progression may be relative to the endocrine treatment [23]. Moreover, PR isoforms may act as ligand-dependent repressors of ER-mediated transcriptional activity and have crosstalk with estrogen signaling [24–26]. It has been shown that PR+/ER+ breast cancers account for more than half of the total cases [27]. Based on the expression of ER, PR, and/or HER2, breast cancer is commonly classified into four main subtypes: luminal A, luminal B, HER2-enriched, and basal-like subtypes [28–31]. Luminal-type breast tumors with ER+ and/or PR+ show a positive response to hormone therapy [32, 33]. A previous study showed that PNPO is induced by estradiol and 4-hydroxytamoxifen in T47D and MCF7 cells (both ER+/PR+), but not in MDA-MB-231 (ER-/PR-) cells [34], implicating that PNPO may correlate with the hormone-related treatment. Triple-negative breast cancer (TNBC), characterized by the low expression of PR, ER, and HER2, is often resistant to chemotherapy. The alteration of hormonal receptor status, such as an increase of PR-B by PNPO-shRNA in the current study, may increase the hormone responsiveness in hormonal therapy.

Our data showed that PNPO was regulated by a lncRNA MALAT1 and a miRNA miR-216b-5p. It has been shown that lncRNAs can act as sponges to bind miRNA through the ceRNA mechanism [35, 36], including MALAT1 [37]. It was reported that MALAT1 is increased in breast cancer tissues and is involved in several cellular processes, including transcriptional and post-transcriptional regulation [11, 38]. Our dual-luciferase reporter assay demonstrated that MALAT1 bound to miR-216b-5p, the latter bound to PNPO 3′-UTR, through the MRE mechanism. MiR-216b has been reported to be a tumor suppressor and have an important role in human cancer progression [39]. Dysregulated expression of miR-216b was found in cancer and may affect cancer cell proliferation and invasion through reciprocal action with other genes [40–42]. The present study exhibited the down-regulation of miR-216b-5p and the up-regulation of PNPO in IDC tissues. Interference of miR-216n-5p impaired the effect of MALAT1 on the regulation of PNPO expression. Combining the results from the dual-luciferase reporter assay, these data indicated that MALAT1 was an upstream regulator of miR-216b-5p and acted as a ceRNA sponge of miR-216b-5p to regulate the expression of PNPO.

**Figure 12:**
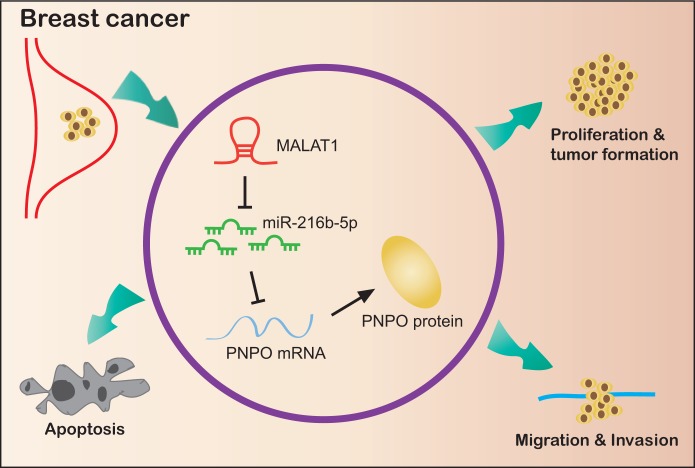
**Schematic illustration of a regulatory mechanism of the MALAT1/miR-216b-5p/PNPO axis in breast ductal cancer.** MALAT1 and PNPO are up-regulated, while miR-216b-5p is down-regulated, in breast cancer cells. The overexpression of MALAT1 mediates PNPO expression by competingly binding to miR-216b-5p, leading to the release of PNPO from the miR-216-5p interaction that avoids the PNPO degradation or translational inhibition. Suppression of PNPO can inhibit breast cancer cell proliferation, migration, invasion and colony formation, and promote breast cancer cell apoptosis.

In summary, we demonstrate for the first time that PNPO and MALAT1 are up-regulated, while miR-216b-5p is down-regulated, in human breast cancer. PNPO is correlated with the overall survival of patients with metastasis at the later stage. The overexpression of MALAT1 mediates PNPO expression by competingly binding to miR-216b-5p, leading to the release of PNPO from the miR-216-5p interaction that avoids the PNPO degradation or translational inhibition. Suppression of PNPO can inhibit breast cancer cell proliferation, migration, invasion and colony formation, and promote cell apoptosis (Figure 12). A regulatory mechanism of the MALAT1/miR-216b-5p/PNPO axis may be important in breast cancer development. Targeting this axis may have therapeutic potential for breast cancer.

## MATERIALS AND METHODS

### Clinical samples and cell lines

This study was approved by the Ethics Committee of Jinshan Hospital. A total of 127 pairs of paraffin-embedded breast IDC tissues and adjacent normal tissues, as well as 105 benign fibroadenomas, were consecutively collected from 2012 to 2016 for IHC study. The follow-up of patients was from January, 2012 to January, 2019. Fresh breast tissues of normal, benign fibroadenomas, and IDC were used for mRNA and protein extraction. All tissues from patients without neoadjuvant therapies such as hormonal therapy, chemotherapy or radiotherapy were immediately frozen in liquid nitrogen after surgery and stored at -80°C for subsequent use. Human non-cancerous cell line MCF-12A and cancerous cell lines MCF-7 and MDA-MB-231 were obtained from American Type Culture Collection (ATCC, Manassas, VA, USA). MCF-12A cells were cultured in DMEM/F12 (Sigma-Aldrich, Cat# D84378, Saint Louis, MO, USA) with 5% FBS (HAO YANG BIOLOGICAL, Cat# TBD15HT, Tianjin, China), 10 μg/ml insulin (Wanbang Pharma, Cat#H19994040, Xuzhou, China), 500 ng/ml hydrocortisone (Shenggong, Cat# A610506, Shanghai, China), and 10 ng/ml EGF (Sigma-Aldrich, Cat# E9644). MCF-7 and MDA-MB-231 cells were cultured in RPMI1640 (Sigma-Aldrich, Cat# R8758) with 10% FBS. The cells were incubated and maintained in a humidified atmosphere of 5% CO_2_ at 37°C.

### Pathological assessment and immunohistochemical staining

All the breast tissue specimens underwent pathological examination after surgery in the Department of Pathology, Jinshan Hospital. The final diagnosis including histological grades and tumor stages were made by experienced pathologists according to the World Health Organization (WHO) classification. Paraformaldehyde-fixed paraffin-embedded tissues were sectioned, deparaffinized and dehydrated. Tissue antigen was retrieval with a citrate buffer (pH 6.0, 15 min), followed by blocking endogenous peroxidase activity with 3% H_2_O_2_ and 10% goat serum, respectively, for 30 min at room temperature. Rabbit anti-PNPO primary antibody (dilution 1:400; Sigma) was added to the slides and incubated overnight at 4°C. HRP-conjugated goat anti-rabbit secondary antibody (dilution 1:500; Shenggong) was added and incubated for 1 h at room temperature, followed by counterstaining the tissue with hematoxylin. The results were determined using a staining index (SI) as described previously [4].

### Real-time quantitative reverse transcription polymerase chain reaction (qRT-PCR)

Total RNA was isolated from clinical specimens or cell lines using the TRIzol reagent (TaKaRa Biotechnology Co., Ltd., Cat#9109, Dalian, Liaoning, China). cDNA was synthesized using a First-Strand Synthesis Kit (Roche Diagnostics, Cat# 04896866001, Indianapolis, IN, USA). qRT-PCR was performed using a FastStart Universal SYBR Green Master (ROX, Roche Diagnostics, Cat# 04707516001) on an ABI7300 platform (Applied Biosystems, Foster City, CA, USA). The expression of mRNAs and MALAT1 was normalized to GAPDH, while miRNA was normalized to U6. The sequences of specific primers are listed in Supplementary Table 5, part of which was designed according to previous reports [43]. The data were calculated with the 2-ΔΔCt method [44].

### Western blot analysis

Total protein was extracted with RIPA buffer (Thermo Fisher Scientific, Cat# 89900, Rockford, IL, USA) supplemented with 10% AEBSF (Shenggong, Cat# C510022) and phosphatase inhibitor cocktail (KeyGen Biotech Co, Ltd, KGP602, Nanjing, Jiangsu, China). Protein concentration was determined using a BCA Kit (Thermo Fisher Scientific, Cat# 23227, Rockford, IL, USA). A total of 20 µg protein was separated on 10% sodium dodecyl sulfate-polyacrylamide gel and transferred to a 0.45 μm polyvinylidene fluoride membrane (Millipore, Cat# IPVH00010, Billerica, MA, USA). The membrane was blocked with 5% non-fat milk or 5% BSA for 1 h at room temperature and incubated overnight at 4°C with primary antibodies diluted in blocking buffer: rabbit-anti GAPDH (1:5000, Cat# D110016, Shenggong), rabbit-anti PNPO (1:2000; Cat# SAB1411034, Sigma), rabbit-anti caspase3, Bax, Bcl2, ERa, PR, and HER2 (1:1000; Cat #9662, #2772, #15071, #8644, #8757, #2165, respectively; Cell Signaling, Danvers, MA, USA) and mouse-anti β-actin (1:5000; Cat# 60008-1-Ig, Proteintech Group, Inc., Wuhan, Hubei, China). Then, the membrane was incubated with an HRP-conjugated goat anti-rabbit (1:5000; Cat# D110058, Shenggong) or mouse (1:5000; Cat# KGAA37, Keygen Biotech, Nanjing, Jiangsu, China) secondary antibody for 1 h at room temperature and the signal was detected using chemiluminescence substrate (Millipore, Cat# WBKLS0100). The OD value of the target band was normalized to GAPDH or β-actin.

### Enzyme-linked immunosorbent assay (ELISA)

The blood samples were allowed to clot for 30 min before centrifugation for 15 minutes at 1000 x *g* and serum was stored at − 80°C for use. The concentrations of PNPO and COL5A1 (another potential biomarker associated with breast cancer reported previously [45]) were measured according to the instructions of the ELISA kits (Cat#: HG2376, Cat# HG0369, TSZ biological Trade Company, Californi, USA). The sensitivity range of PNPO and COL5A1 was 25–2000 pg/ml and 3.5 −280 μg/L, respectively. Each sample was diluted five times and measured twice for the average.

### Lentiviral shRNA transduction

Control and human PNPO short hairpin RNA (PNPO-shRNA, shPNPO) were constructed using pHY-LVKD5.1 RNAi lentivirus (contained GFP) (Hanyin Biotechnology, Shanghai, China). MCF-12A, MCF-7, and MDA-MB-231 cells were infected with PNPO-shRNA or control shRNA using X-tremeGENE™ siRNA Transfection Reagent (Cat# 4476093001, Roche Applied Science) at a concentration of 30 multiplicity of infection (MOI) with 8 µg/ml polybrene according to the manufacturer’s instructions. After 24 h post-incubation, the medium was replaced and 2 µg/ml of puromycin was added for cell selection. The transduction efficiency was analyzed by fluorescence microscopy. Knockdown of PNPO expression at mRNA and protein levels in infected cells was confirmed by Western blot and qRT-PCR, respectively.

### Transfection of miRNA mimics, inhibitors, and siRNAs

RNA oligos (mimics and inhibitors) and their corresponding scrambled negative control (NC) were purchased from GenePharm (Shanghai, China). Briefly, After seeding cells into a 6-well plate at 2×10^5^/well for 12 h, cells were transfected with miRNA-216b-5p mimics or inhibitors at a concentration of 50 nM or 100 nM, respectively, using XtremeGENE Transfection Reagent (Roche Applied Science) and incubated for 48 h. Small interfering RNA (siRNA) and NC for MALAT1 knockdown were performed in the same way.

### Cell viability assay

Cell viability was measured by the Cell Counting Kit 8 (CCK8) assay (Dojindo, Cat# LB625, Tokyo, Japan) following the manufacturer’s instruction. Transfected cells were seeded in a 96-well plate at 3×10^3^/well and cultured for 48 h. The optical density was detected at 450 nm absorption.

### Cell cycle analysis

For DNA profile (cell cycle) analysis, cells were seeded into a 6-well plate at 4×10^5^/well and incubated for 24 h until 80% confluence. Cells were starved for 24 h in serum-free medium before cells harvest. Then, cells were washed with cold PBS and resuspended in 70% cold ethanol for fixation and stored in the freezer for 2 h. After removing ethanol, cells were washed and incubated with a cell cycle solution (BD Biosciences, Cat# 550825, San Diego, CA, USA) on ice for 10 min. The cell population in different phases was determined by flow cytometry (Beckman).

### Wound-healing assay

PNPO-shRNA-expressing or NC cells were plated on 12-well plates at 3×10^5^/well and cultured until subconfluence. The cell monolayer was scratched with a sterile 200-µl plastic tip and washed with PBS to remove the cell debris. The cells were continuously incubated in RPMI 1640 medium supplemented with 10% FBS for 48 h. Images of the plates were captured under a microscope at 0 and 48 h after scratching. The width of the gap was photographed and analyzed with CellSens Life Science Imaging Software (OLYMPUS, Tokey, Japan).

### Cell invasion assays

For the invasion assay, 1×10^5^ cells were suspended in serum-free medium and seeded in the upper chamber of a 24-well Transwell (Corning, NY, USA) with 8-μm pore polycarbonate membrane, which was precoated with Matrigel (Corning) at a final concentration of 250 µg/ml. Medium with 10% FBS was added to the bottom chamber. After incubation for 48 h, the invaded cells on the lower chamber membrane surface were fixed in methanol for 20 min, stained in 0.1% crystal violet (Cat# V5265, Sigma-Aldrich Co.) for 30 min and photographed. The number of invading cells was counted with ImageJ software (National Institutes of Health, Bethesda, MD, USA).

### Colony formation assay

NC or PNPO-shRNA cells (5×10^3^) were seeded in a 60-mm dish and cultured for 14 days. After washing, cells were fixed with 4% paraformaldehyde for 20 min and stained with 0.1% crystal violet for 30 min. Colony numbers were photographed under a light microscope and analyzed with ImageJ software.

### Apoptosis assay

Annexin V-APC/PI apoptosis detection system (Annexin V-APC, Cat# 550474; PI, Cat# 51-66211E; Binding buffer, Cat# 51-66121E; BD Biosciences, San Diego, CA, USA) was utilized to assess apoptotic cells. Briefly, cells were harvested and subsequently washed with cold PBS. Then, cells were resuspended in 500 μL 1× binding buffer supplemented with 5 μL of Annexin V-APC and 5 μL PI for 15 min on ice in the dark before flow cytometry analysis (Beckman, German).

### Dual-luciferase reporter assay

The 3′-UTR fragment of PNPO that contains a predicted binding site of miR-216b-5p was amplified from genomic DNA and inserted into the reporter vector pmirGLO (Promega, Cat#E1330, Madison, WI, USA). The corresponding mutated PNPO was synthesized simultaneously and verified by DNA sequencing. HEK-293T cells were cultured in 24-well plates and cotransfected with 0.5 µg wild-type PNPO or mutated PNPO reporter plasmid and 50 nM miR-216n-5p mimics or negative controls for 48 h. Cells were harvested and the Luc-Pair™ Duo-Luciferase Assay Kit (GeneCopoeia, Cat#LF002, Rockville, MD, USA) was used to determine luciferase activities following the manufacturer’s instructions. Renilla luciferase activity was applied for standardization. The same approach was used for the interaction between MALAT1 and miR-216b-5p.

### Statistical analysis

All results are presented as the mean ± SEM or SD from at least three independent experiments as indicated. Statistical analyses were carried out using GraphPad Prism 6 (La Jolla, CA, USA) and SPSS v22.0 (Chicago, IL, USA) softwares. Quantitative analyses were applied with 2-tailed Student’s t-test, one-way analysis of variance, Chi-square test, or Fisher exact test for comparison. ROC curves were analyzed by calculating the AUC. To investigate the synergetic effect of PNPO and COL5A1 in serum, a logistic regression model was applied to display AUC changes. Log-rank (Mantel-Cox) test was used for the survival rate curve analysis. The subgroup analyses for OS with HR and 95% CIs were conducted by the univariate and multivariate analyses using the Cox proportional hazard regression model. A statistically significant value was considered at P < 0.05.

### Ethics Approval

The project was approved by the Ethics Committee of Jinshan Hospital, Fudan University (JYLLKY-2017-11-01).

## Supplementary Material

Supplementary Figure

Supplementary Tables
