# Supplementary material for: Pyridoxine 5′-phosphate oxidase is correlated with human breast invasive ductal carcinoma development
Source: Aging (Albany NY). 2019 Apr 14;11(7):2151–76. doi: 10.18632/aging.101908 (PMC6503878; doi:10.18632/aging.101908)
Supplement: Supplementary Figure [file aging-11-101908-s001.pdf]

SUPPLEMENTARY FIGURE

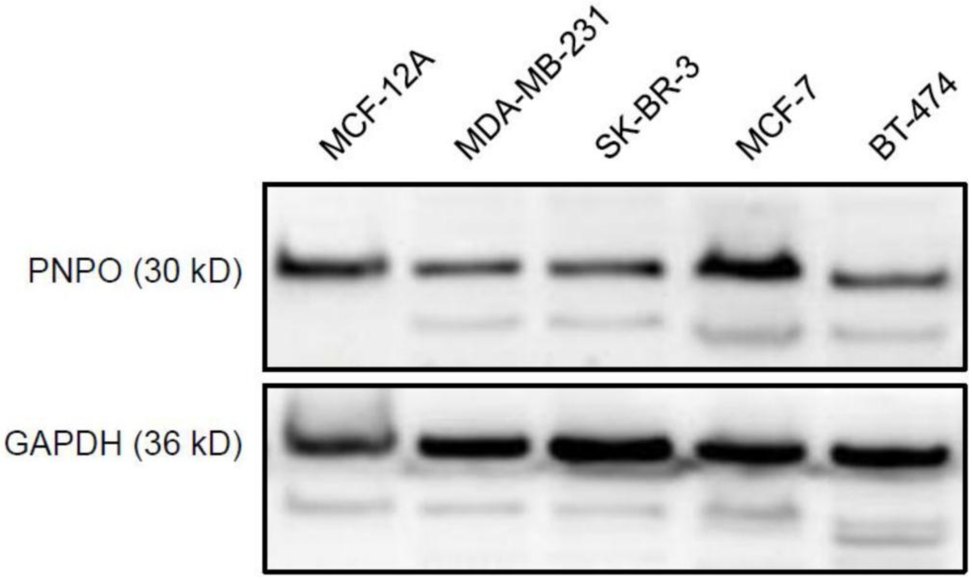

Cell line / Expression	MCF-12A	MDA-MB-231	SK-BR-3	MCF-7	BT-474
ER	-	-	-	+	+
PR	-	-	-	+	+
HER2	-	-	+	-	+

Supplementary Figure 1. Human breast non-cancerous cell line (MCF-12A) and breast cancer cell lines (MDA-MB-231, SK-BR-3, MCF-7, and BT-474) were used. MDA-MB-231 and SK-BR-3 are hormone-sensitive breast cancer cell lines; MCF-7 and BT-474 are hormone-insensitive breast cancer cell lines (BMC Genomics. 2016; 17 Suppl 7: 525). Upper panel shows the expression of PNPO protein in five breast cells detected by Western blot. Lowe panel shows the expression features of five breast cells. ER, estrogen receptor; PR, progesterone receptor; HER2, human epidermal growth factor receptor-2; -, negative expression; +, positive expression.
