# Supplementary material for: Pyridoxine 5′-phosphate oxidase is correlated with human breast invasive ductal carcinoma development
Source: Aging (Albany NY). 2019 Apr 14;11(7):2151–76. doi: 10.18632/aging.101908 (PMC6503878; doi:10.18632/aging.101908)
Supplement: Supplementary Tables [file aging-11-101908-s002.pdf]

SUPPLEMENTARY TABLES

Supplementary Table 1. Correlation between PNPO protein expression and clinicopathological features of patients with IDC.

Clinicopathological features	n = 127	PNPO expression		Chi-square	P-value
		Low (n = 56)	High (n = 71)		
Age (years)				0.4881	0.485
≤60	88	37	51		
>60	39	19	20		
LN metastasis				1.2457	0.264
No	75	30	45		
Yes	52	26	26		
Tumor size (mm)				0.3193	0.572
≤20	58	24	34		
>20	69	32	37		
Histological grade				1.5934	0.451
1	7	4	3		
2	80	32	48		
3	40	20	20		
Clinical stage				*	0.778
I	41	17	24		
II	58	26	32		
III	27	13	14		
IV	1	0	1		
ER expression				4.1994	0.040
Low	71	37	34		
High	56	19	37		
PR expression				3.5099	0.061
Low	84	42	42		
High	43	14	29		
HER2 expression				0.1515	0.697
Low	52	24	28		
High	75	32	43		
Ki-67 positive				2.4037	0.121
≤10%	56	29	27		
>10%	71	27	44		

Average age: 54.85±12.39 years (mean ± standard deviation) (Range: 30-85). The expression of PNPO protein was detected by immunohistochemistry. A chi-square test was applied for the comparison of PNPO correlated with the clinicopathological features, except * Fisher's exact test. IDC, invasive ductal carcinoma; LN, lymph node; ER, estrogen receptor; PR, progesterone receptor; HER2, human epidermal growth factor receptor-2; n, number of cases.

Supplementary Table 2. Comparisons of the levels of PNPO concentration in serum associated with clinicopathologic features.

Variable	Cases (Total n = 30)	Average PNPO (pg/mL)	P-value
Grade			0.799
1+2	25	508.23 ± 16.95	
3	5	497.09 ± 48.50	
LN metastasis			0.548
No	18	498.40 ± 51.96	
Yes	12	518.34 ± 29.34	
ER expression			0.842
Low	19	508.86 ± 20.93	
High	11	502.09 ± 25.20	
PR expression			0.767
Low	25	508.54 ± 17.79	
High	5	495.56 ± 38.19	
HER2 expression			0.407
Low	14	491.99 ± 19.06	
High	16	518.96 ± 24.86	
Ki-67 positive			0.674
≤10%	20	496.67 ± 28.71	
>10%	10	511.23 ± 19.49	

Concentration is presented as the mean ± the standard error of the mean. LN, lymph node; ER, estrogen receptor; PR, progesterone receptor; HER2, human epidermal growth factor receptor-2; n, number of cases.

Supplementary Table 3. Subgroup analysis for the overall survival of patients with breast IDC (univariate analysis).

Variables	Total cases (TD%)	Univariate analysis HR (95% CIs)	P-value
PNPO expression			
Low	56 (1.786)	*	
High	71 (5.634)	0.307 (0.035~2.662)	0.284
Age (years)			
Age low/PNPO low	37 (2.703)	*	
Age low/PNPO high	51 (5.882)	0.549 (0.055~5.442)	0.608
Age high/PNPO low	19 (0.000)	32.876 (0.000~5.354E+9)	0.719
Age high/PNPO high	20 (5.000)	0.416 (0.026~6.644)	0.535
LN metastasis			
Non-metastasis/PNPO low	30 (0.000)	*	
Non-metastasis/PNPO high	45 (0.000)	N/A	
Metastasis/PNPO low	26 (3.846)	0.008 (0.000~2.064E+5)	0.582
Metastasis/PNPO high	26 (15.385)	0.017 (0.000~24.715)	0.273
Tumor size (mm)			
Size≤20mm/PNPO low	24 (0.000)	*	
Size≤20mm/PNPO high	34 (0.000)	N/A	
Size>20mm/PNPO low	32 (3.125)	0.018 (0.000~1.808E+5)	0.624
Size>20mm/PNPO high	37 (10.811)	0.026 (0.000~311.523)	0.446
Histological grade			
Grade (1+2)/PNPO low	36 (0.000)	*	
Grade (1+2)/PNPO high	51 (7.843)	0.023 (0.000~100.771)	0.377
Grade (3)/PNPO low	20 (5.000)	0.008 (0.000~2.064E+5)	0.582
Grade (3)/PNPO high	20 (0.000)	N/A	
Clinical stage			
Stage (I+II)/PNPO low	66 (0.000)	*	
Stage (I+II)/PNPO high	33 (0.000)	N/A	
Stage (III+IV)/PNPO low	13 (7.692)	0.000 (0.000~5.399E+40)	0.852
Stage (III+IV)/PNPO high	15 (26.667)	0.001 (0.000~2173.800)	0.363
ER expression			
ER low/PNPO low	37 (2.703)	*	
ER low/PNPO high	34 (5.884)	0.394 (0.035~4.370)	0.448
ER high/PNPO low	19 (0.000)	43.709 (0.000~8.228E+8)	0.658
ER high/PNPO high	37 (5.405)	0.670 (0.059~7.650)	0.748
PR expression			
PR low/PNPO low	42 (2.381)	*	
PR low/PNPO high	42 (4.762)	0.534 (0.047~6.076)	0.613
PR high/PNPO low	14 (0.000)	32.366 (0.000~5.354E+9)	0.719
PR high/PNPO high	29 (6.897)	0.342 (0.031~3.791)	0.382
HER2 expression			
HER2 low/PNPO low	24 (0.000)	*	

HER2 low/PNPO high	28 (7.143)	0.034 (0.000~5.930E+4)	0.645
HER2 high/PNPO low	32 (3.125)	0.017 (0.000~1.661E+5)	0.619
HER2 high/PNPO high	43 (4.651)	0.019 (0.000~1.912E+3)	0.500
Ki-67 positive			
≤10%/PNPO low	29 (3.448)	*	
≤10%/PNPO high	27 (7.407)	0.449 (0.041~4.963)	0.514
>10%/PNPO low	27 (0.000)	46.890 (0.000~6.949E+8)	0.648
>10%/PNPO high	44 (4.545)	0.921 (0.080~10.629)	0.947

Subgroup analyses for overall survival were conducted by the univariate analysis using the Cox proportional hazard regression model. IDC, invasive ductal carcinoma;TD, tumor-related death; HR, hazard ratio; CIs, confidence intervals; LN, lymph node; ER, estrogen receptor; PR, progesterone receptor; HER2, human epidermal growth factor receptor-2; * referent category; N/A, non-applicable because the number of TD case is zero in both referent and subject categories.

Supplementary Table 4. Multivariate analyses for the prognostic factor of PNPO associated with the overall survival of patients with IDC in univariate analyses.

Variables	Multivariate analysis HR (95% CIs)	P-value
PNPO		
Low	*	
High	0.514 (0.057~4.645)	0.553
LN metastasis		
No	*	
Yes	0.000 (0.000~3.250E+137)	0.963
Clinical stage		
Early stage (I+II)	*	
Later stage (III+IV)	0.000 (0.000~5.186E+135)	0.944

The prognostic effect of PNPO was performed by multivariate analyses using the Cox proportional hazard regression model with factors being significantly associated with the outcome of patients with IDC in univariate analyses. IDC, invasive ductal carcinoma; HR, hazard ratio; CIs, confidence intervals; LN, lymph node; * referent category.

Supplementary Table 5. Sequences of primers, siRNA, shRNA, mimics and inhibitor used in the present study.

	Sequence (5′-3′)
**Primer:**	
PNPO-F	TTGAGGAGACTCATCTGACC
PNPO-R	GTTAGTGAAGAAGCGGAAGC
ER-F	ACTTGCTCTTGGACAGGAACCA
ER-R	CAAACTCCTCTCCCTGCAGATT
Total PR-F	AGTTCTTTGCTGACAAGTCTTAATCAAC
Total PR-R	TCGAAAACCTGGCAATGATTTAG
PR-B-F	TGCTGGACAGTGTCTTGGAC
PR-B-R	CACCAAGAGCTGGTGACCTC
HER2-F	AGGTGGTGCAGGGAAACCTG
HER2-R	CAGGGGTGGTATTGTTCAGC
MALAT1-F	AGCCAGTGCGATTTGGTGA
MALAT1-R	TCCTCCGTGTGGTTGCCAA
GAPDH-F	TGCACCACCAACTGCTTAGC
GAPDH-R	GGCATGGACTGTGGTCATGAG
β-actin-F	GTTGTCGACGACGAGCG
β-actin-R	GCACAGAGCCTCGCCTT
miR-216b-5p-F	GAAATCTCTGCAGGCAAATGTG
miR-216b-5p-R	GTGCAGGGTCCGAGGT
miR-216b-5p-RT	GTCGTATCCAGTGCAGGGTCCGAGGTATTCGCACTGGATACGACtcacat
U6-R	CTCGCTTCGGCAGCACA
U6-F	AACGCTTCACGAATTTGCGT
**MALAT1-siRNA**	
Sense	GGCAAUGUUUUACACUAUUTT
Antisense	AAUAGUGUAAAACAUUGCCTA
**PNPO-shRNA**	
Sense	gatccGACTGGCTCTATGAGAGACTTCAAGAGAGTCTCTCATAGAGCCAGTCTTTTTTg
Antisense	aattcAAAAAAGACTGGCTCTATGAGAGACTCTCTTGAAGTCTCTCATAGAGCCAGTCg
**miR-216b-5p**	
Mimics-sense	AAAUCUCUGCAGGCAAAUGUGA
Mimics-antisense	ACAUUUGCCUGCAGAGAUUUUU
NC-sense	UUCUCCGAACGUGUCACGUTT
NC-antisense	ACGUGACACGUUCGGAGAATT
Inhibitor	AAAUCUCUGCAGGCAAAUGUGA
Control of inhibitor	CAGUACUUUUGUGUAGUACAA

F, forward primer; R, reverse primer; RT, reverse transcription; NC, negative control.
